# Therapeutic effects of curcumin on the functional disturbances and oxidative stress induced by renal ischemia/reperfusion in rats

**Published:** 2015

**Authors:** Houshang Najafi, Saeed Changizi Ashtiyani, Sayed Abolhasan Sayedzadeh, Zeynab Mohamadi yarijani, Sajad Fakhri

**Affiliations:** 1 *Medical Biology Research Center, Kermanshah University of Medical Sciences, Kermanshah, Iran *; 2 *Department of Physiology, School of Medicine, Arak University of Medical Sciences, Arak, Iran*; 3 *Department of Nephrology, Emam Reza Hospital, Kermanshah University of Medical Sciences, Kermanshah, Iran*; 4 *School of pharmacy, Kermanshah University of Medical Sciences, Kermanshah, Iran*

**Keywords:** *Curcumin*, *Acute renal failure*, *Oxidative stress*, *Ischemia/Reperfusion*

## Abstract

**Objective:**

Curcumin has anti-inflammatory and antioxidative properties. The objective of this study was to investigate the therapeutic effects of curcumin on functional disturbances, oxidative stress, and leukocyte infiltration induced by renal ischemia/reperfusion (I/R).

**Materials and Methods:**

Animals were randomly divided into 9 groups. The groups with 24-h reperfusion consisted of sham-24h, I/R-24h, and three I/R groups treated with curcumin at 10, 20, or 30 mg kg^-1^, i.p. after the ischemic period. The 72-h reperfusion groups also included Sham-72h, I/R-72h, I/R treated with curcumin at single dose of 20 mg kg^-1^, i.p., and I/R group which received three doses of curcumin at 20 mg kg^-1^, i.p., consecutively. Renal functional injury was assessed by measuring serum creatinine and urea-nitrogen concentrations. Oxidative stress was evaluated by assessment tissue malondialdehyde (MDA) and the ferric reducing/antioxidant power (FRAP) levels. Moreover, renal tissue leukocyte infiltration was measured by histopathology examination.

**Results:**

Ischemia/reperfusion resulted in a significant increase in serum concentration of creatinine, urea-nitrogen, tissue MDA level, and leukocytes infiltration as well as reduced FRAP level. Treatment with curcumin in 24-h reperfusion groups could only lead to a significant change in the levels of MDA and FRAP. However, in 72-h reperfusion groups, curcumin was able to correct all functional disturbances, oxidative stress, and leukocytes infiltration with more effectiveness in groups that received three doses of curcumin.

**Conclusion:**

The administration of curcumin during 72-h reperfusion following 30 minutes of ischemia can decrease renal oxidative stress and leukocytes infiltration as well as improve kidney function. However, during first 24-h reperfusion, curcumin only decreased oxidative stress.

## Introduction

*Curcuma longa* (turmeric) is an herbaceous plant that its rhizomatous parts have been widely used as food coloring and flavoring substance. Curcuma contains various compounds such as carbohydrates, proteins, fats, fiber, and about 3-5% of curcuminoids. Curcuminoids include curcumin (70%), demethoxycurcumin (17%), bisdemethoxycurcumin (3%), and 10% cyclocurcumin (Jayaprakasha et al., 2002 ▶; Trujillo et al., 2013 ▶). Curcumin (diferuloylmethane) is insoluble in water and ether, but is soluble in alcohol, glacial acetic acid, and olive oil (Ammon and Wahl, 1991 ▶). Due to various properties of curcumin, it has been widely studied in biological systems.

Curcumin exert its anti-inflammatory (Srimal and Dhawan, 1973 ▶; Ammon et al., 1993 ▶; Kuhad et al., 2007 ▶) and antioxidant (Masuda et al., 1999 ▶; Tirkey et al., 2005 ▶) effects by inhibiting 5-lipoxygenase and reducing different cytokines including TNF-α, IL-1β, and INF-γ. Kuhad and colleagues (2007) ▶ showed that use of curcumin 2 days before and 3 days after the administration of cisplatin significantly decreased renal damage, oxidative stress, and systemic inflammation in rats. Tirkey et al. (2005) ▶ reported that curcumin was able to reduce oxidative stress and also improve renal function and tissue damages resulting from chronic use of cyclosporine.

Moreover, curcumin has been shown to have an inhibitory effect against the apoptosis by inhibiting TGF-β and caspase-3 (Awad and El-Sharif, 2011 ▶). Indeed, many studies have shown that curcumin has a protective effect against functional disturbances, tissue damages, oxidative stress, and inflammation caused by I/R in the kidney (Bayrak et al., 2008 ▶; Awad and El-Sharif, 2011 ▶; Rogers et al., 2012 ▶) as well as other organs (Kim et al., 2012 ▶; Okudan et al., 2013 ▶).

Recently, curcumin has also been considered for its clinical and therapeutic applications. Most importantly, curcumin has been used for the treatment of rheumatoid arthritis (Deodhar et al., 1980 ▶), postoperative inflammation (Satoskar et al., 1986 ▶), idiopathic orbital inflammation (Lal et al., 2000 ▶), Alzheimer's disease, multiple myeloma (Hatcher et al., 2008 ▶), pancreatic and colon cancer (Lao et al., 2006 ▶; Kanai, 2014 ▶), and chronic renal failure (Trujillo et al., 2013 ▶).

 The most important damages caused by renal I/R are vascular endothelium and tubular epithelium damages that result in the development of oxidative stress and interstitial inflammation (Thadhani et al., 1996 ▶; Kribben et al., 1999 ▶; Clarkson et al., 2008 ▶). Therefore, this study aimed to investigate the therapeutic effects of curcumin on functional disturbances and oxidative stress caused by I/R in the rat model. 

## Materials and Methods

This experimental study was conducted on 63 male Wistar rats weighing 200-250 g. During the experiment, the internationally accepted guidelines for the care and use of laboratory animals in biologic research was observed (978-0-309-15401-7). All animals in this experiment were kept under controlled condition of temperature (22-24 °C), 12 hours light/dark cycles, and were provided with standard rat food and water ad libitum. 

**Ischemia and reperfusion induction**

 After anesthesia with ether (Moosavi et al., 2010 ▶; Moosavi et al., 2011 ▶), a midline abdominal incision was made through linea alba using electrocautery and the renal artery and vein were simultaneously clamped for 30 min, bilaterally. At the end of ischemia, the surgery area was stitched and curcumin or its solvent (ethilic alcohol) was injected to animals intraperitoneally. Then, animals were returned to cages with free access to food and water. All the tools used in this procedure were sterilized by autoclave or Deconex.

 Throughout the course of surgery, the animal body temperature was measured via a rectal probe. By using a heat lamp and a heating plate, the animal body temperature was maintained within the range of 37 °C±1 (Jafarey et al., 2014 ▶; Najafi et al., 2014 ▶). Animals examined in this study were randomly divided into 9 groups (n=7 in each group): sham 24-h group (Sham-24h), ischemia and 24-h reperfusion group (I/R-24h), I/R-24h group receiving curcumin at 10 mg/kg, i.p. (I/R + C10-24h), I/R-24h group which received curcumin at 20 mg/kg, i.p. (I/R + C20-24h), I/R-24h group which received curcumin at 30 mg/kg, i.p. (I/R + C30-24h), sham group with a 72-hour reperfusion period (Sham-72h), ischemia and 72-h reperfusion group (I/R-72h), I/R-72h group which received a 20 mg/kg, i.p. dose of curcumin (I/R + C20(1) -72h), and I/R-72h group that received three doses of 20 mg/kg, i.p. of curcumin every 24 hours (I/R + C20(3) -72h) during 72-h reperfusion period.

**Evaluation of renal function**

 After 24 or 72 hours of reperfusion period, animals were anesthetized and the surgical area was opened. Then, a blood sample was taken from descending aorta and its serum was used for creatinine and urea-nitrogen concentration measurements. MDA and FRAP levels were measured in the right kidneys and the left kidneys were also used to prepare slides and stained with hematoxylin-eosin (H & E) for pathological examination. At the end of experiment, the animals were sacrificed by deep anesthesia (Najafi et al., 2014 ▶). 

 Creatinine and urea-nitrogen concentrations were measured by an autoanalyzer (Technicon, RA-1000, USA). To determine leukocytes infiltration in the kidney, sections stained with H & E were prepared and cortical regions, outer medulla, and inner medulla were separately studied using a light microscope. Number of leukocytes in 20 microscopic fields (area of each field was 0.14 mm^2^) were counted by a pathologist and the average was calculated as the number in a cubic millimeter (Ysebaert et al., 2000 ▶).

The evaluation of oxidative stress was performed by measuring MDA, which is the end product of lipids peroxidation by reactive oxygen species (ROS) and also FRAP based on Ohkawa (1979) ▶ and Benzie (1999) ▶, respectively (Ashtiyani et al., 2013 ▶; Changizi Ashtiyani et al., 2013 ▶). For the measurement of MDA, the kidney tissue was homogenized using a tissue homogenizer in phosphate buffer and then acetic acid (20%), thiobarbituric acid (0.8%), and sulfate deododecyl sodium (8.1%) were added to all of the test tubes. After heating the resultant suspension at 95 °C for 60 minutes for interaction with malondialdehyde, the optical absorbance of pink complex was measured at wave length of 523 nm using a spectrophotometer (SpectroLab 7500 UV, England). For the measurement of FRAP, a fresh FRAP reagent was prepared. This reagent contained 2.5 mL of 10-mmol tripyridyl-s-triazine solution in 40 mmolar hydrochloric acid, 2.5 mL of ferric chloride, and 2.5 mL of 0.3-mol acetate buffer. After that, 50 μL of the tissue extract was added to each of the test tubes and the level of FRAP was determined by measuring light absorption in 593-nm wave length. All reagents were purchased from Sigma-Aldrich (St Louis, MO, USA).

**Statistical analysis**

 Results were shown as mean ± SEM. The one-way ANOVA followed by Duncan's post hoc test was used for the intergroup comparison of all measured parameters, and LSD test was used to determine the exact p value. All data were analyzed by SPSS-18 statistical software and p<0.05 was considered as significant level.

## Results

**The effect of curcumin on renal functional disturbances**

**Figure 1:**
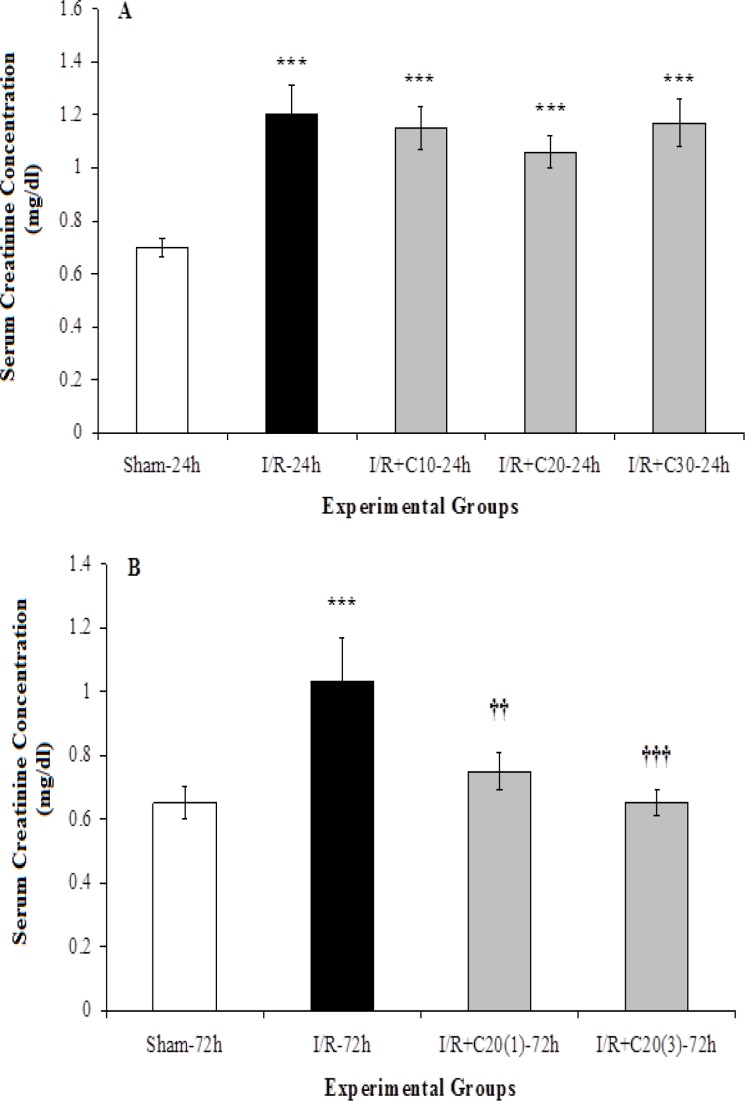
Serum creatinine concentration in (A) rats with renal ischemia and 24-hour reperfusion that received ethanol (I/R-24h) or curcumin with different doses (I/R+C), or sham surgery (sham-24h); and (B) with renal ischemia and 72 hours of reperfusion that received ethanol (I/R-72h) or curcumin with single 20 mg kg^-1^ dose (I/R+C20(1)-72h) or three doses (I/R+C20(3)-72h), and sham surgery (sham-72h) animals. ***p<0.001 in comparison with the sham group.^ ††^p<0.01,^ †††^p<0.001 001 in comparison with the I/R group

Applying a half-hour bilateral renal ischemia and 24 hours reperfusion increased serum creatinine concentration (p<0.001) in the I/R group compared to the sham animals (Figure 1-A). However, in this study, the administration of three different doses of curcumin could not significantly reduce the serum creatinine concentration in I/R+C-24h groups in comparison with the I/R-24h group. Among the groups treated with different doses of curcumin in 24-hour reperfusion period, no significant difference was observed in serum creatinine concentration.

Serum creatinine concentration in I/R-72h group was also considerably higher than that in sham-72h group (p<0.001). Administration of curcumin at a single dose or three doses of 20 mg/kg of body weight over a period of 72-hours of reperfusion (I/R+C20(1)-72h and I/R+C20(3)-72h groups) could significantly reduce serum creatinine compared to the I/R-72h group with a higher reduction in the group that received three doses of curcumin (p<0.01 vs. p<0.001). There was no significant difference in serum creatinine concentration between I/R+C20(1)-72h and I/R+C20(3)-72h groups and the sham group (Figure 1-B).

**Figure 2:**
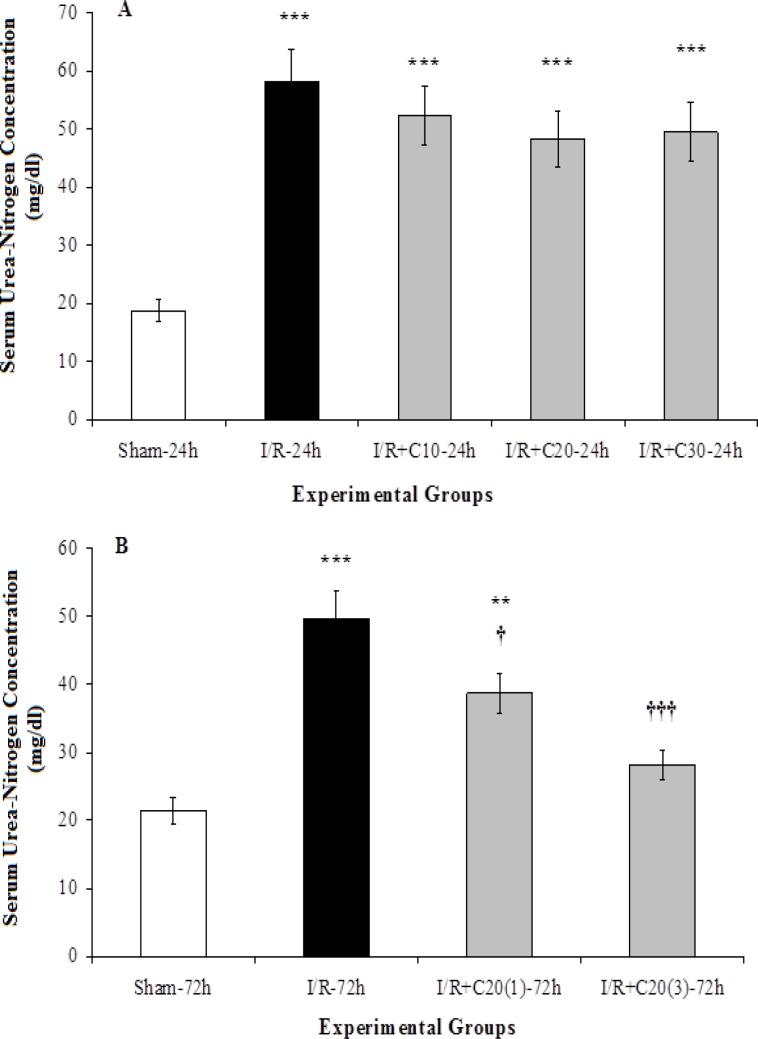
Serum urea-nitrogen concentration in (A) rats with renal ischemia and 24 hours of reperfusion that received ethanol (I/R-24h) or curcumin with different doses (I/R+C), or sham surgery (sham-24h); and (B) with renal ischemia and 72 hours of reperfusion that received ethanol (I/R-72h) or curcumin with single 20 mg kg-1 dose (I/R+C20(1)-72h) or three doses (I/R+C20(3)-72h), and sham surgery (sham-72h) animals. *p<0.05, **p<0.01, ***p<0.001 in comparison with the sham group. †p<0.05, ††p<0.01, †††p<0.001 001 in comparison with the I/R group

As figure 2-A shows, serum urea-nitrogen concentration in sham-24h group was 18.7 ± 1.9 mg/dl which increased to 58.2 ± 5.6 mg/dl in I/R-24h group (p<0.001). Curcumin administration at three different doses could not lead to a significant decrease in serum urea-nitrogen concentration in groups with 24-hours reperfusion period in comparison to I/R-24h group. 

The serum concentration of urea-nitrogen in I/R-72h was significantly higher than the sham-72h group (p<0.001). Although a single dose of curcumin could significantly lower urea-nitrogen concentration in I/R+C20(1)-72h group as compared to I/R-72h group (p<0.05), it was still higher than that the related sham animals (p<0.01). 

Three doses of curcumin during 72 hours of reperfusion resulted in a significant decrease in serum urea-nitrogen concentration in the I/R+C20(3)-72h group compared to I/R-72h (p<0.01). Moreover, there was no significant difference between I/R+C20(3)-72h group in comparison with the related sham group (Figure 2-B).

**Effect of curcumin on oxidative stress**

**Figure 3:**
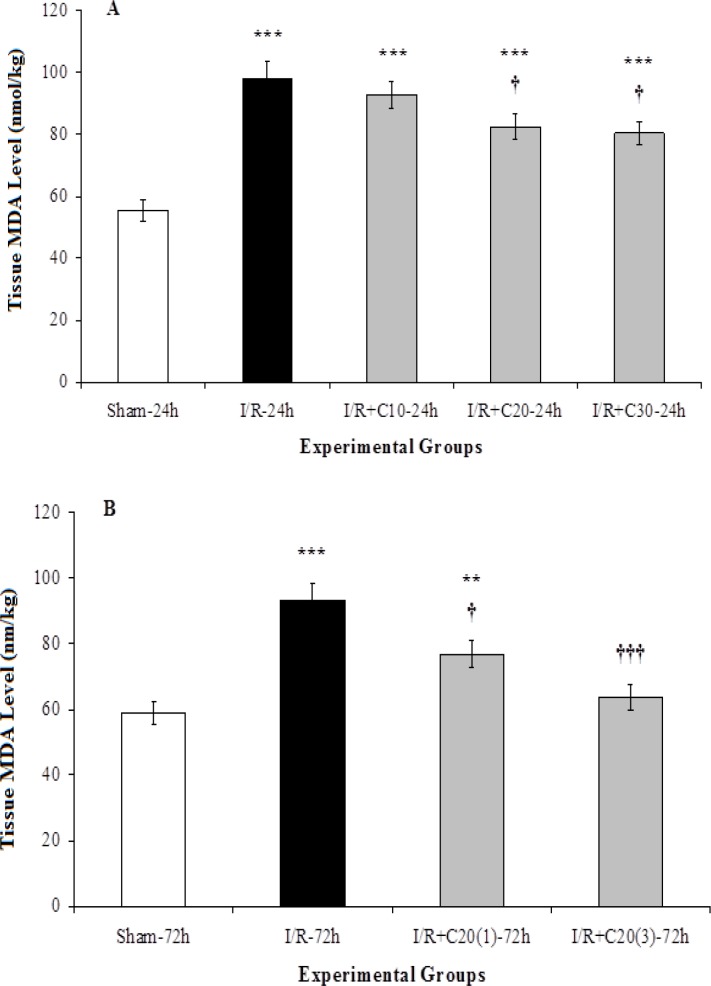
Tissue MDA per gram kidney weight in (A) rats with renal ischemia and 24 hours of reperfusion that received ethanol (I/R-24h) or curcumin with different doses (I/R+C), or sham surgery (sham-24h); and (B) with renal ischemia and 72 hours of reperfusion that received ethanol (I/R-72h) or curcumin with single 20 mg kg^-1^ dose (I/R+C20(1)-72h) or three doses (I/R+C20(3)-72h), and sham surgery (sham-72h) animals. *p<0.05, **p<0.01, ***p<0.001 in comparison with the sham group.^ †^p<0.05,^ ††^p<0.01,^ †††^p<0.001 001 in comparison with the I/R group

Figure 3-A shows that MDA level in kidney tissue of sham-24h group was 55.46 ± 3.54 nanomole per gram of kidney weight which after 30 minutes of renal ischemia and 24 hours of reperfusion in I/R-24h group significantly increased by 78% in comparison to related sham group (p<0.001). Although the use of curcumin could significantly reduce tissue MDA levels in I/R+C20-24h and IR+C30-24h groups compared to I/R-24h (p<0.05), they were still higher than that in sham-24h group (p<0.001). MDA level in I/R-72h was also significantly higher than sham-72h (p<0.001). 

Application of curcumin in single and three doses of 20 mg/kg body weight during the 72-hour period of reperfusion could reduce MDA level in the I/R+C20(1)-72h and I/R+C20(3)-72h groups compared to the I/R-72h group (p<0.05 vs. p<0.001). So that there was no significant difference in tissue MDA level between I/R+C20(3)-72h group and its related sham group (Figure 3-B).

**Figure 4:**
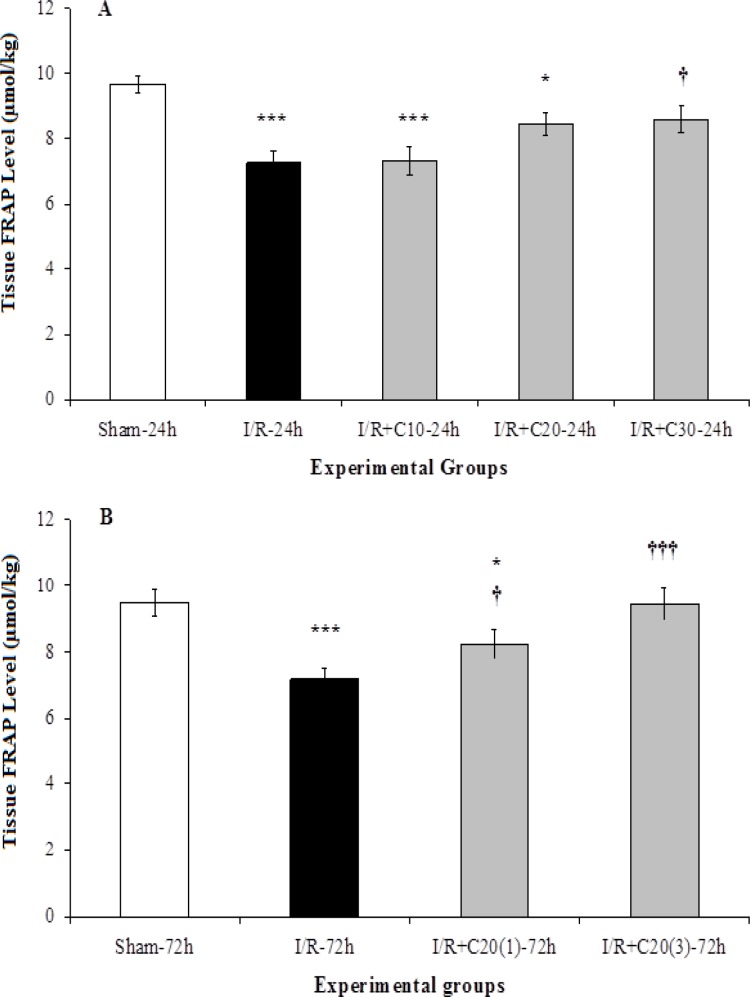
Tissue FRAP per gram kidney weight in (A) rats with renal ischemia and 24 hours of reperfusion that received ethanol (I/R-24h) or curcumin with different doses (I/R+C), or sham surgery (sham-24h); and (B) with renal ischemia and 72 hours of reperfusion that received ethanol (I/R-72h) or curcumin with single 20 mg kg^-1^ dose (I/R+C20(1)-72h) or three doses (I/R+C20(3)-72h), and sham surgery (sham-72h) animals. *p<0.05, **p<0.01, ***p<0.001 in comparison with the sham group.^ †^p<0.05,^ ††^p<0.01,^ †††^p<0.001 001 in comparison with the I/R group

Our results also showed that I/R led to severe kidney tissue FRAP reduction (p<0.001) in I/R-24h group compared to its value in the sham-24h group (Figure 4-A). Although the administration of three different doses of curcumin could increase FRAP level, this change was only significant in the I/R+C30-24h group in comparison with the I/R-24h group (p<0.05). FRAP level in the I/R-72h group was significantly lower than sham-72h group (p<0.001). Applying curcumin in both single dose and three doses was able to significantly increase FRAP level in I/R+C20(1)-72h and I/R+C20(3)-72h groups compared to the I/R-72h group, though was more effective in the group receiving three doses of curcumin (Figure 4-B).

**The effect of curcumin on leukocyte infiltration**

**Figure 5:**
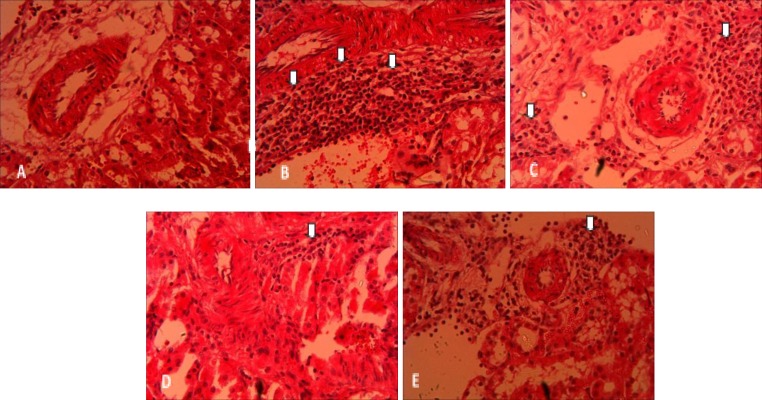
Light microscopic photographs of renal cortex for representing leukocytes infiltration (arrow) from rats in sham-24h (A), I/R-24h (B), I/R+C10-24h (C), I/R+C20-24h (D), and I/R+C30-24h (E) groups; Hematoxilin-Eosin staining, magification×400

**Figure 6:**
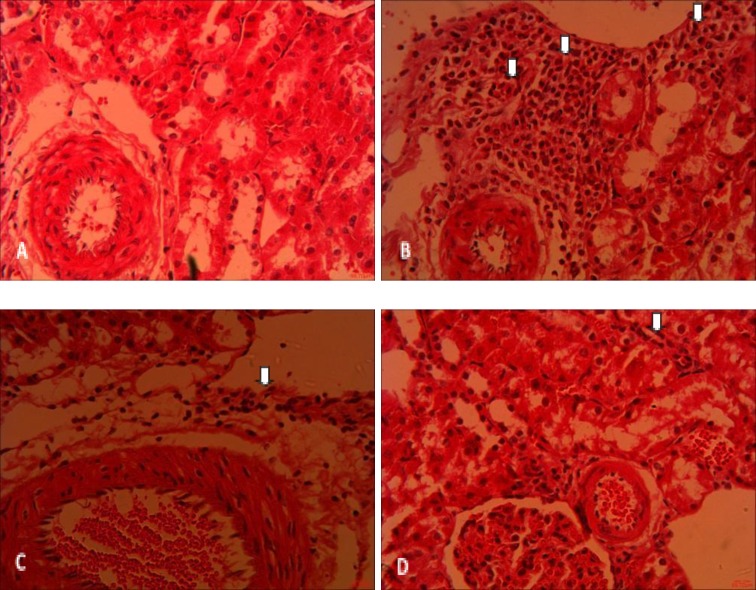
Light microscopic photographs of renal cortex for representing leukocytes infiltration (arrow) from rats in sham-72h (A), I/R-72h (B), I/R+C20(1)-72h (C), and I/R+C20(3)-72h (D) groups; Hematoxilin-Eosin staining, magification×400

Images that are related to leukocyte infiltration in the renal interstitium are shown in Figures 5 and 6. The number of leukocytes in the sham-24h group was 1-3 leukocytes per square millimeter of the renal interstitium. The renal ischemia and reperfusion could increase the level of leukocyte infiltration up to 42 times in I/R-24h group in comparison to the corresponding sham group (p<0.001). As shown in the Figures 5-C, D, and E using three different curcumin doses in rats with 24-hour reperfusion period reduced leukocyte infiltration in the renal interstitium.

The leukocyte infiltration in the I/R-72h group was also significantly higher than the corresponding sham group (p<0.001). Following curcumin administration, leukocyte infiltration decreased by 8.5 times in the I/R+C20(1)-72h group (p<0.01) in comparison to the I/R-72h group. However, in the I/R+C20(3)-72h group, its amount reached that of the sham group. 

## Discussion

The results of present study showed that intraperitoneal administration of curcumin during 72-h reperfusion after bilateral renal ischemia could partially improve functional disturbances, oxidative stress, and leukocyte infiltration in the kidneys. Moreover, it demonstrated that effect of three successive doses were more than a single dose. However, curcumin treatment in the I/R- 24h group could only improve renal oxidative stress. Serum creatinine and urea-nitrogen concentrations in I/R group highly increased, possibly because of a sharp decline in the glomerular filtration rate (GFR). Several studies have demonstrated that renal I/R by disturbing the balance between the production of vasoconstrictors such as adenosine and endothelin and vasodilators including nitric oxide and prostaglandins increase the renal vasoconstriction (Baek et al., 1975 ▶; Kribben et al., 1999 ▶). Moreover, increased adhesion molecules and sticking of leukocytes, platelets, and red blood cells to vascular endothelium lead to intravascular congestion, causing a permanent decrease in total renal blood flow during the reperfusion period. Decreased renal blood flow, increased pressure of Bowman's capsule, and increased back leakage through damaged epithelial layer of tubules also result in a severe decline of GFR in acute kidney injury (Kribben et al., 1999 ▶).

In our study, applying ischemia/reperfusion caused an increase in renal tissue MDA and also a reduction in FRAP levels in I/R group, which was in line with results of previous studies (Chatterjee et al., 2003 ▶; Valko et al., 2007 ▶). It has been shown that I/R, in addition to activating ROS-producing enzymes, decreases the enzymes of antioxidant defense system (Winther et al., 1999 ▶).

Experimental studies have shown that I/R also leads to a rapid release of pro-inflammatory cytokines (Slofstra et al., 2007 ▶) and lipid peroxidation (Granger, 1988 ▶), which in turn causes inflammation and oxidative stress. Inflammation also leads to the activation of leukocytes that directly or through producing ROS, proxy nitrite (ONOO^-^), and eicosanoids causes damage to the endothelial and tubular cells. In addition, leukocytes produce and secrete the myeloperoxidase enzyme which catalyzes H_2_O_2_ to hypochlorous acid (HOCl^-^) which is a more active oxidant (Clarkson et al., 2008 ▶).

The use of curcumin in this study led to a partial recovery of renal function and also reduced oxidative stress and leukocyte infiltration resulted from I/R. Although this decrease may be due to reduced renal damages (Prodjosudjadi et al., 1995 ▶), some investigators have shown that curcumin also directly inhibits chemokines (Xu et al., 1997 ▶). 

Moreover, it has been suggested that curcumin leads to induction of heme-oxygenase-1 (HO-1) enzyme expression in renal epithelial cells through Nrf2/ARE pathway (Balogun et al., 2003 ▶), which is also a protective mechanism against oxidative stress. It has also been shown that curcumin through the stimulation of HO-1 can inhibit the TNF-α induced ICAM-1 expression, thereby inhibiting leukocytes infiltration (Youn et al., 2013 ▶). 

Rogers and colleagues (2012) ▶ also showed that curcumin decreases expression of thioredoxin-interacting protein (TXNIP). Ischemia/reperfusion by up-regulating TXNIP and thereby inhibiting thioredoxin leads to the induction of oxidative stress. On the other hand, curcumin stimulates the transcription of genes which induce the expression of antioxidant systems such as glutathione peroxidase, glutathion-S-transferase, catalase, and superoxide dismutase (Rogers et al., 2012 ▶; Trujillo et al., 2013 ▶).

Therefore, it can be concluded that curcumin possibly reduces leukocyte infiltration and functional disturbances in the rat kidney via supporting the kidney against oxidative stress.
